# An Innovative Method of Resizing the Nipple-Areola Complex in Subcutaneous Mastectomy: A Report of Three Cases

**DOI:** 10.7759/cureus.83403

**Published:** 2025-05-03

**Authors:** Amina I Abubakar, Yakubu Sabo, Samuel Okpechi, Chukwuma E Chijioke

**Affiliations:** 1 Surgery, College of Health Sciences, University of Abuja, Abuja, NGA; 2 Surgery, University of Abuja Teaching Hospital, Abuja, NGA; 3 Surgery, Federal Medical Centre Abuja, Abuja, NGA; 4 Surgery, Garki Hospital Abuja, Abuja, NGA

**Keywords:** case report, gender-affirming, nipple areola complex, subcutaneous mastectomy, surgery

## Abstract

Gender-affirming mastectomy (GAM), also known as ‘top surgery,’ is one of the pivotal procedures undergone by trans males. Most procedures described, such as subcutaneous mastectomy, address breast volume reduction adequately, but resizing the nipple and areola is still a challenge.

We describe an innovative method for resizing the nipple-areola complex, reducing the scar burden, and preserving innervation and acceptable male aesthetics.

## Introduction

It can be quite devastating for those who wish for a masculine identity to have a feminine type of breast. Gender-affirming mastectomy (GAM), also known as ‘top surgery,’ has been used as a masculinization surgery to give trans males a cosmetically appealing flat chest [1,2]. Masculinizing surgeries are similar to regular gynecomastia surgeries. GAM ranges from subcutaneous mastectomy, reduction mammoplasty with preservation of the nipple-areola complex, to mastectomy with or without preservation of the nipple-areolar complex [2,3]. These procedures aim to improve chest contour, reduce chest measurement, reposition the nipple-areola complex, and minimize scarring.

GAM has a role in the treatment of patients with feminizing features caused by disorders of sex development (DSD) and medication-induced gynecomastia. Though GAM addresses the projection of the breast, it does not adequately address the size of the nipple-areola complex (NAC) in patients who have, in addition to large breasts, large nipples, wide areolas, and, in some instances, nipple protrusion. The average male NAC is usually higher and more laterally positioned than that of females [4], measuring 2-3 cm in diameter with nipples about 2.6 cm high [5,6].

The goal of applying this technique was to preserve innervation to the NAC while achieving an aesthetically appealing masculine NAC. This report aims to describe an innovative method of resizing the NAC in three patients - one with gynecomastia and two with disorders of sex development - who had an extended, semi-circular, circumareolar, subcutaneous mastectomy with resection of a sector of the NAC.

## Case presentation

Procedure

**Figure 1:**
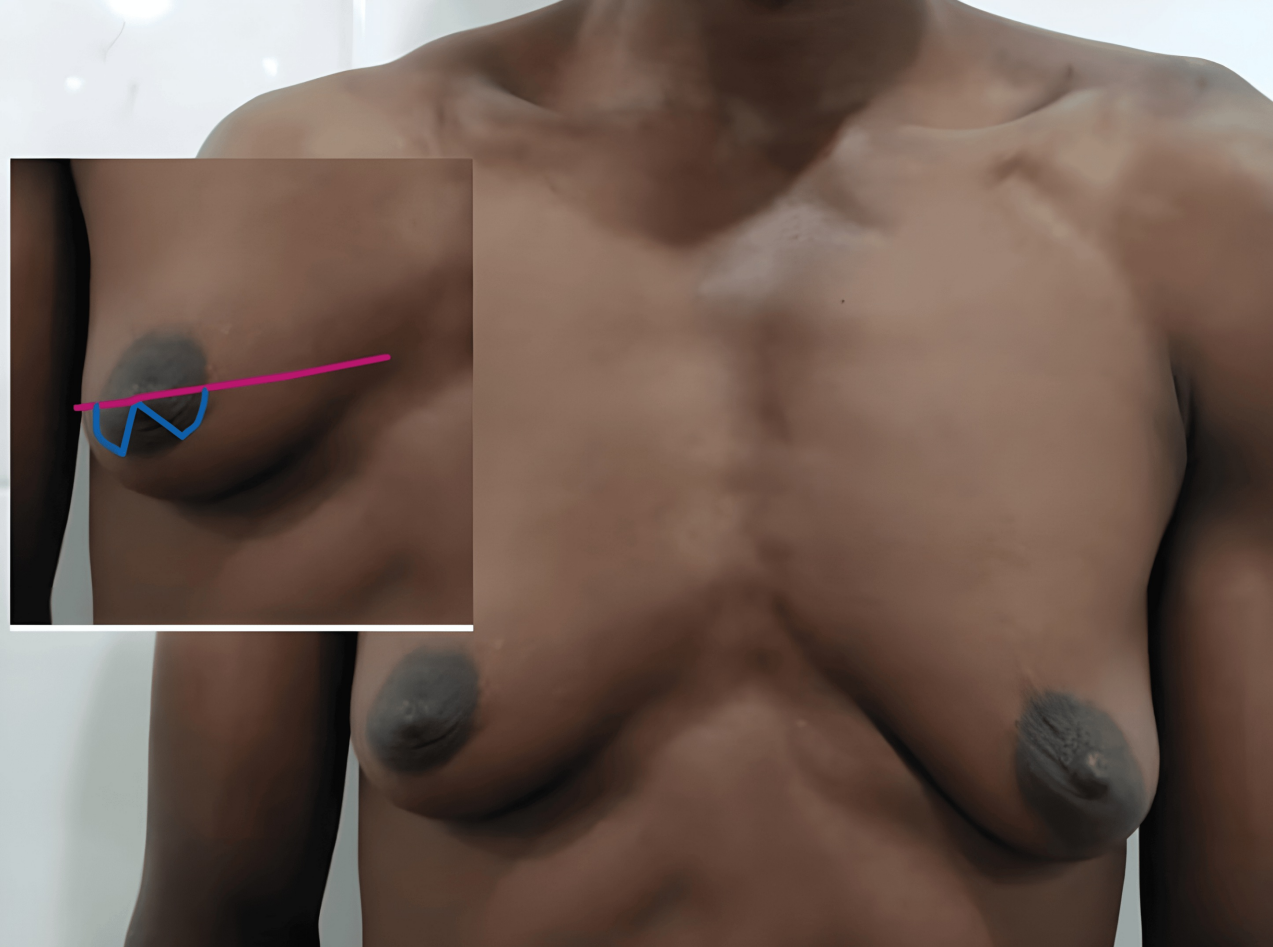
Preoperative markings

The patient is marked in a standing position with arms by the side in a relaxed stance. The transverse diameter of the NAC is marked to define it. The semi-circumareolar marking is extended bilaterally, stopping short of the vertical one-quarter of the NAC (Figure 1). From the center of the nipple, an inverted V is marked, extending the lower quarter sector of the NAC.

All the surgeries are done in the theater under general anesthesia, with the patients placed in the supine position. The initial incision is along the lower diameter of NAC and then to the nipple sector marking. The incision is deepened to the subcutaneous layer, excising the NAC sector under the V, and the rest of the subcutaneous mastectomy is completed through this incision.

**Figure 2:**
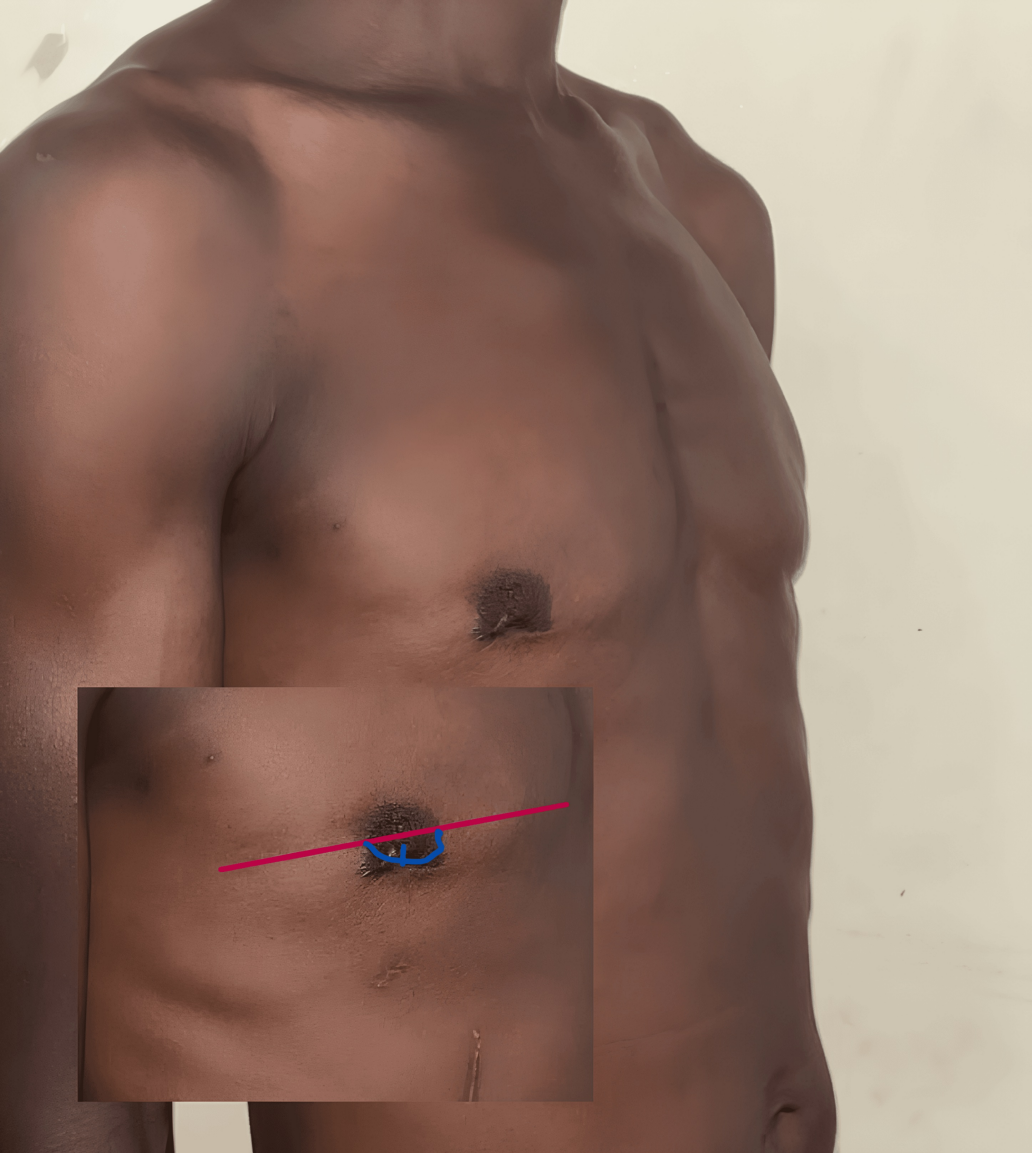
Postoperative skin closure

The vertical edges of the V incision are brought together and closed in the midline. The NAC is reduced by a quarter of its size. An active drain is inserted, and the rest of the NAC incision is closed with subcuticular absorbable stitches (Figure 2).

Each patient is fitted with a compression bandage from the theater, given adequate analgesia, and has a 48-hour dose of intravenous broad-spectrum antibiotics.

The patients are discharged when the drains are no longer active (3-5 days). All patients are instructed to minimize the use of the upper limbs for two weeks after surgery.

Case 1

This was a 70-year-old man diagnosed with adenocarcinoma of the prostate, initially being managed with regular prostate-specific antigen (PSA) surveillance. He developed an accelerated rise in PSA and was started on an androgen-deprivation therapy drug - bicalutamide, and a gonadotropin-releasing hormone agonist - goserelin. He subsequently developed gynecomastia with feminization of the NAC. In his words, his breasts were now similar to his wife’s, and he could not go shirtless. He desired a masculine chest with a male NAC.

On examination, he was a fit, elderly gentleman with a B-cup-sized breast, soft and slightly tender to the touch. The NAC diameter was 8 cm, and the nipples were 1 cm wide and projected 1 cm above the breast.

**Figure 3:**
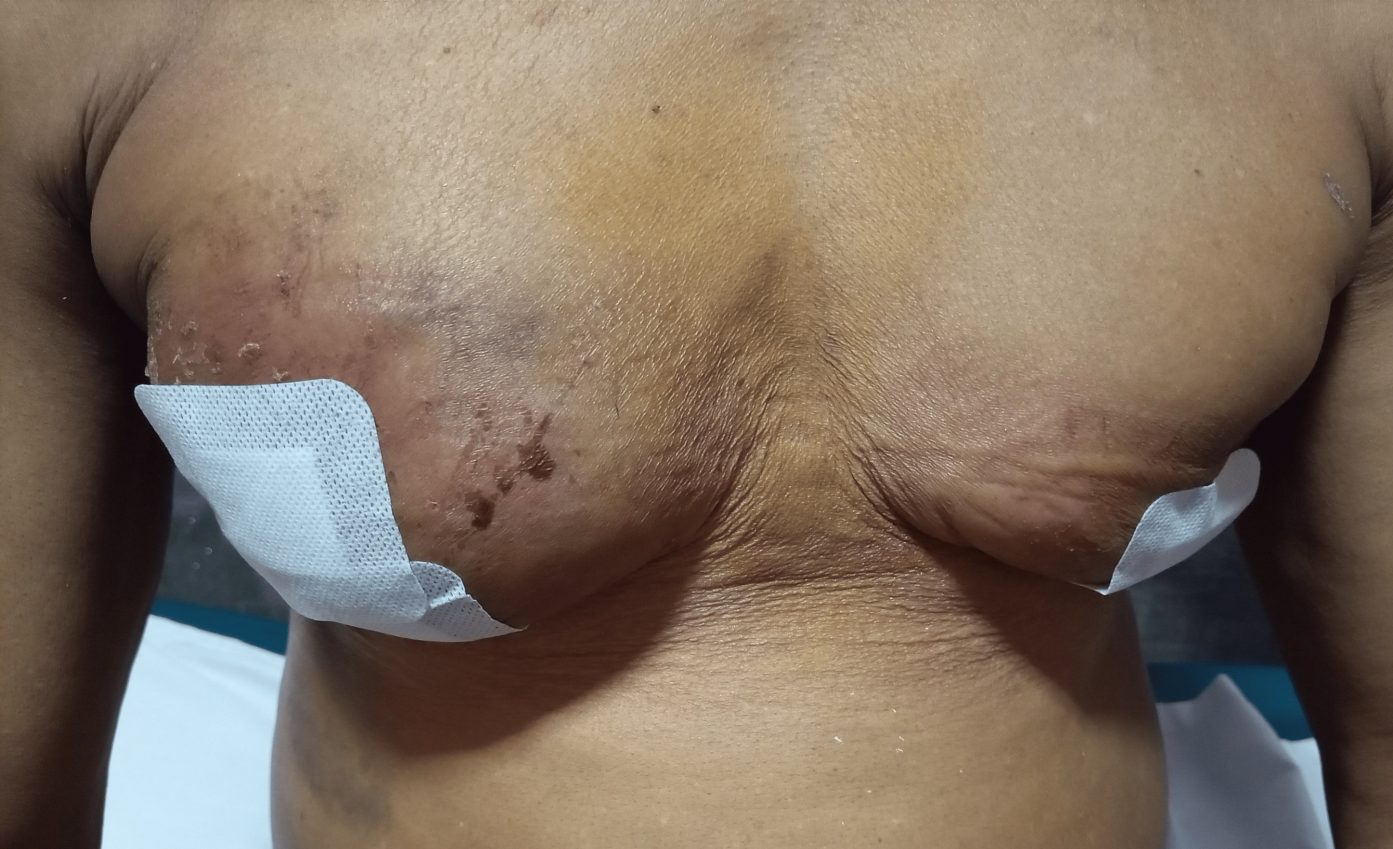
Postoperative hematoma in a 70-year-old man on the seventh postoperative day

The patient had the procedure as prescribed, and he developed a hematoma at home on the seventh day postop while trying to prepare a starchy local meal (Figure 3). It was evacuated as an office procedure. The remaining healing period was uneventful.

Case 2

**Figure 4:**
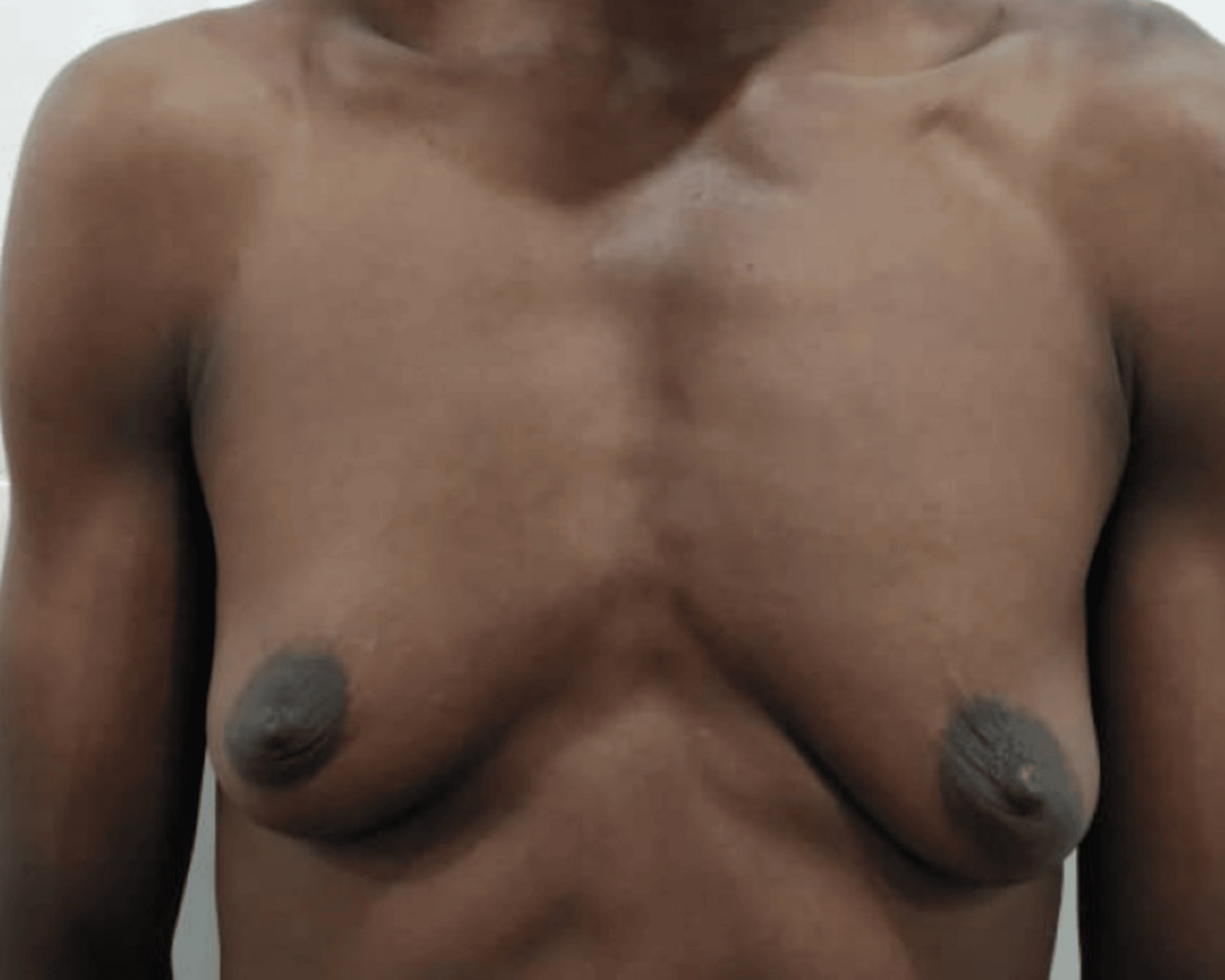
Preoperative image of a 40-year-old DSD with fully formed female breasts DSD: disorders of sex development

**Figure 5:**
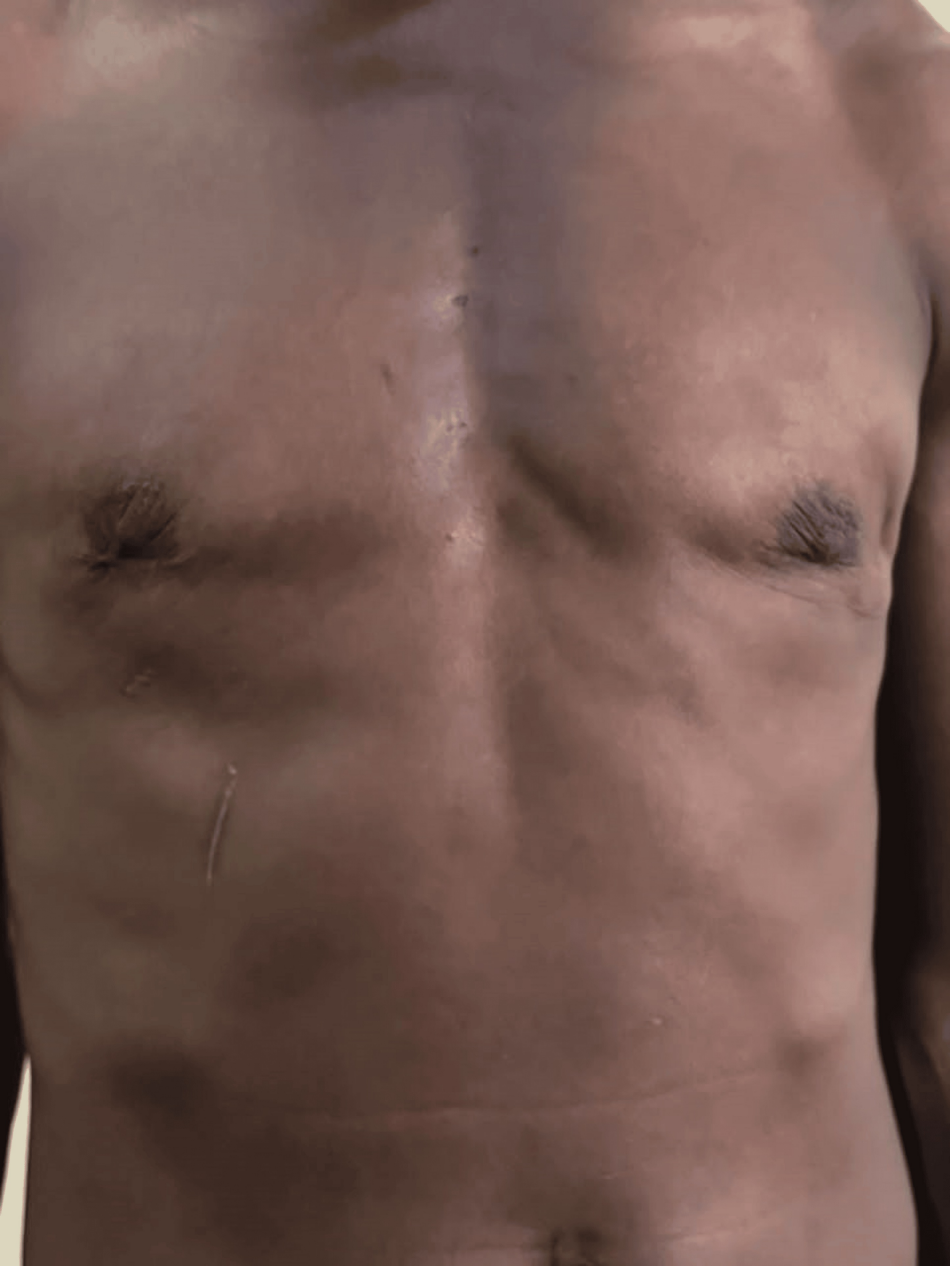
Postoperative image of Case 2 twelve months after surgery

This was a 40-year-old DSD patient with an XXY karyotype, raised male. He wished to retain the male gender identity. He presented with well-formed female breasts. He had a hysterectomy and oophorectomy as well as a subcutaneous mastectomy at the same surgery. Figure 4 shows the preoperative picture, and Figure 5 shows the twelfth-month postoperative results.

Case 3

**Figure 6:**
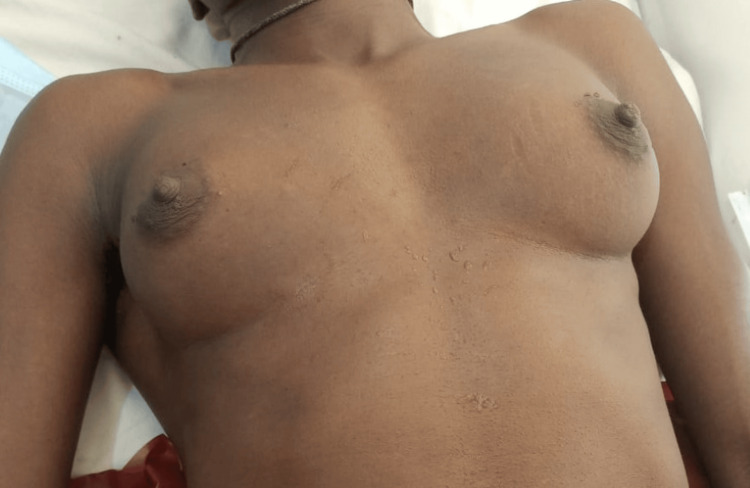
Pre-operative picture of a 35-year-old DSD patient with fully formed breasts DSD: disorders of sex development

**Figure 7:**
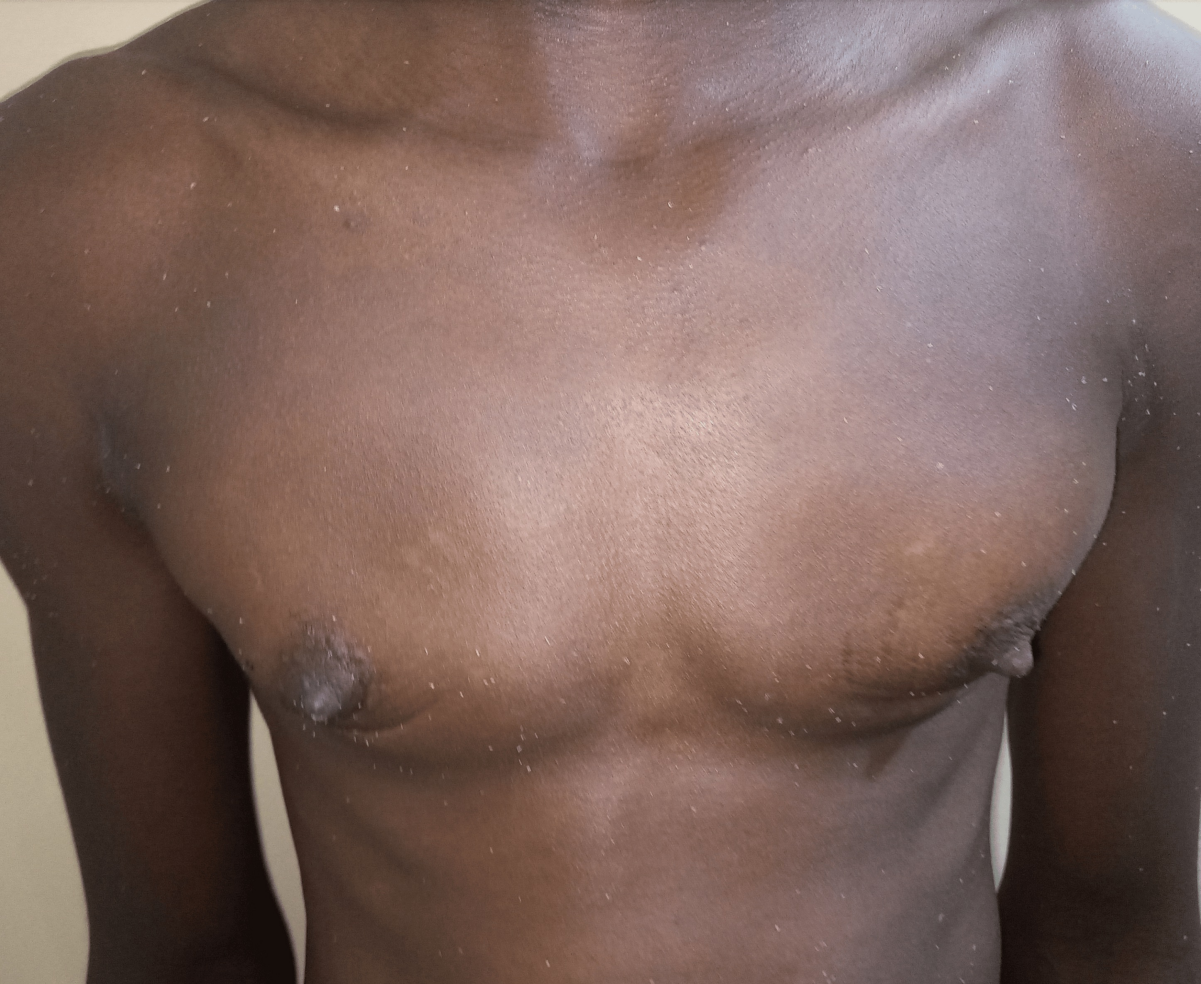
Patient in Case 3 twelve months after surgery

A 35-year-old DSD patient with an XXY karyotype, raised male, wished to retain the male gender identity. He was referred by the gynecologist after undergoing a hysterectomy and oophorectomy. He presented with well-formed female breasts. Figure 6 shows a preoperative picture, and Figure 7 shows the twelfth-month postoperative results.

## Discussion

Gynecomastia is any condition in which the male breast volume increases; it usually does not feminize the NAC [7]. Surgical interventions differ from GAM because they do not address the feminized NAC [5].

There are various procedures described, but the semicircular circumareolar approach was used in all three patients. The male NAC is usually higher and more laterally positioned than the female. Bustos et al. found in their review that the NAC placement is according to the surgeon’s discretion on the table [8]. The lead author and surgeon made this decision in all three cases. The aim was to achieve a smaller, more ovoid NAC that suits the patients’ aesthetic goals.

Resizing of the NAC can be done as a second-stage procedure to preserve blood supply [1]; all three patients had a one-stage procedure. The initial motivation for a one-stage procedure was to reduce the financial burden on the patients who pay out of pocket in our region of practice.

None of the patients experienced complications such as necrosis, scarring, or loss of sensation in the NAC, though only one patient experienced hematoma, which is a common complication of this procedure [2]. 

There have been many attempts to produce a more masculine NAC and address nipple projection for patients undergoing subcutaneous GAM [8,9]. Our procedure is an innovative one that we believe adds to the options available to the surgeon and patient undergoing subcutaneous GAM. This procedure offers a drastic reduction in the diameter of the NAC without the need for nipple amputation, another method of reducing the nipple size. We have also found that patients are more satisfied with this method.

## Conclusions

Gender-affirming gynecomastia (GAM) is a life-changing experience for males, in order to restore or confirm their masculinity. The burden of scars that come with "top surgery" sometimes outweighs the satisfaction patients derive from the procedure. Our method of reducing the size of the NAC by a quarter sector ensures a gender-correct size and shape, while reducing the burden of scars. All our patients were satisfied with the surgical outcome.

## References

[1] Song S, Kim EA (2023). Double-incision mastectomy after reduction mammaplasty for persistent gender dysphoria: a case report. Case Reports Plast Surg Hand Surg.

[2] Monstrey S, Selvaggi G, Ceulemans P (2008). Chest-wall contouring surgery in female-to-male transsexuals: a new algorithm. Plast Reconstr Surg.

[3] McTernan M, Yokoo K, Tong W (2020). A comparison of gender-affirming chest surgery in nonbinary versus transmasculine patients. Ann Plast Surg.

[4] Ammari T, Sluiter EC, Gast K, Kuzon WM Jr (2019). Female-to-male gender-affirming chest reconstruction surgery. Aesthet Surg J.

[5] Lindsay WR (1979). Creation of a male chest in female transsexuals. Ann Plast Surg.

[6] Vigneswaran N, Lim J, Lee HJ, Ong WC, Rasheed MZ, Lim TC (2013). A novel technique with aesthetic considerations in female-to-male transsexuals nipple areola complex reconstruction. J Plast Reconstr Aesthet Surg.

[7] Swerdloff RS, Ng JCM (2023). Gynecomastia: etiology, diagnosis, and treatment. Endotext [Internet].

[8] Bustos SS, Kuruoglu D, Yan M (2021). Nipple-areola complex reconstruction in transgender patients undergoing mastectomy with free nipple grafts: a systematic review of techniques and outcomes. Ann Transl Med.

[9] Knox AD, Ho AL, Leung L (2017). A review of 101 consecutive subcutaneous mastectomies and male chest contouring using the concentric circular and free nipple graft techniques in female-to-male transgender patients. Plast Reconstr Surg.

